# Diversity and Life History Traits of Native Weed Communities in Agricultural Areas: A Case Study in Eastern China

**DOI:** 10.3390/biology13090704

**Published:** 2024-09-07

**Authors:** Guoqi Chen, Zeyue Huang, Kai An, Yang Chen, Jiahao Xue

**Affiliations:** Jiangsu Key Laboratory of Crop Genetics and Physiology/Jiangsu Key Laboratory of Crop Cultivation and Physiology, Agricultural College/Research Institute of Rice Industrial Engineering Technology of Yangzhou University, Jiangsu Co-Innovation Center for Modern Production Technology of Grain Crops, Yangzhou University, Yangzhou 225009, China; hh1003741822@163.com (Z.H.); 16638727457@163.com (K.A.); yzuchenyang@163.com (Y.C.); 18205251761@163.com (J.X.)

**Keywords:** family, field survey, herbicide resistance, life history trait, occurrence dominance, species richness, weediness

## Abstract

**Simple Summary:**

Plant invasion represents a significant global ecological challenge, and effective management of invasive species requires robust weed risk assessment systems. These systems predominantly rely on understanding the relationships between life history traits and weed occurrence. In this study, we conducted a survey of native weed communities across 666 field sites in agricultural areas of Yangzhou City, China, covering a longitudinal range of 0.86°, a latitudinal range of 0.84°, and an altitude below 100 m. Our findings reveal that dominant weed species are concentrated within a few families and are associated with higher levels of weediness globally. The key traits of prevalent native weeds include moderate plant height, absence of thorns, non-succulent, erect growth forms or lianas, and adaptability to a range of light conditions, whether mesophytic or xerophytic. Dominant native weeds were frequently annuals, biennials, or perennials with moderate vegetative reproduction abilities and high fertility, possessing seeds of moderate size and fruits without mucilage, conspicuous hairs, thorns, or awns. Additionally, many have developed herbicide resistance. This study is the first to focus on the diversity and life history traits of native weed communities in China, providing valuable insights for refining weed risk assessment and management strategies.

**Abstract:**

Native weeds have a long history of adaptation to local environments. Understanding the relationship between the occurrence of native weeds and their life history traits is crucial for effective weed management and risk assessment of plant invasions. In this study, we surveyed native weed species and their dominance across 666 field sites in agricultural areas of Yangzhou City, China, and each site was 13.3 hectares in area. A total of 287 native weed species were recorded, referring to 63 families, among which 45% were 50–100 cm in plant height and 47% were of an erect life type. In terms of the proportions out of the total native weed occurrence dominance, Poaceae, Compositae, and Fabaceae weeds accounted for 30%, 13%, and 11%; liana and perennials both occupied 32%; and aquatic, hygrophyte, sun plant, and shade plant all occupied < 10%. Additionally, the proportions increased with increasing seed production per plant and with increasing weediness reported worldwide. Native weed groups holding moderate vegetative reproduction abilities, moderate seed sizes, or herbicide resistance showed higher proportions. Moreover, most of the native weeds surveyed were not succulent or thorny plants and did not hold thorns, awns, obvious hairs, or mucilage on their fruits.

## 1. Introduction

Weeds refer to plants with populations that are all or mainly grown in environments disturbed by humans [1]. Among crops, the total global loss due to weeds varied from 7.5% to 10.5% [2]. Weeding is essential for human society to engage in agricultural production, landscape maintenance, biodiversity conservation, and other activities. On the other hand, as a part of biodiversity, weeds are also important resource plants [3]. Many weed species have a series of values, such as medicinal, feeding, edible, ornamental, and breeding resources. Therefore, weediness has been proposed to distinguish the ability or potential of different types of weeds to cause damage to human society. Moreover, understanding how weed communities assemble as a function of biotic and abiotic filters and transform through time has important implications for the sustainable management of agronomic systems [4]. Therefore, field surveys on weed communities are one of the basic works in weed sciences.

Research processes in weediness are also commonly used in the risk assessment of alien invasive plants to guide the introduction of plants [5]. For example, the Australian Weed Risk Assessment System (AWRA) is an effective pre-border screening weed tool that is widely used around the world [6]. This system divides the risk of plant species based on responses to 49 questions, such as species biology and biogeography [7,8]. The American Weed Risk Assessment System (USWRA) was developed using a logistic regression model based on the Australian WRA [9]. In Europe, Weber and Gut (2004) developed a Europe-specific weed risk assessment system (WG-WRA) using a quantitative grading system similar to AWRA [10,11]. These risk assessment systems usually assign values based on a series of crucial traits of specific plants and finally obtain a numerical risk coefficient, which is used to determine the risk of invasion. Therefore, having knowledge of the corresponding relationship between a series of traits and weediness is important for weed management and risk assessment of plant invasions.

Baker [12] first studied the related traits of weediness in 1965 by comparing weeds and non-weeds; he discussed the factors that make a species become weeds and defined the concept of ‘ideal‘ weeds. Many researchers have studied weed-related indicators [13,14], with numerous studies focusing on comparisons between exotic and native weeds [15]. Systematic analysis based on native weed communities may also yield important results. Native weeds have experienced a long history of adaptation in the local areas and have a long-term interaction and adaptation to the local environments. Distributions of native weeds are generally closely related to their adaptive characteristics [16]. Therefore, having knowledge of the relationships between the occurrence of native weeds and their life history traits could be important for weed management and risk assessment of plant invasions.

We conducted systematic field surveys on native weed communities in agricultural areas in Yangzhou City, Jiangsu Province, China, from 2022 to 2023. A total of 666 field sites were randomly selected, and each site was 13.3 ha. in area. We investigated the native weed species and their relative dominance, respectively, in each site. As a case study based on field survey data on native weed occurrence, this study aimed to reveal the important life history traits relating to weediness in agricultural areas.

## 2. Materials and Methods

### 2.1. Investigating Area

Yangzhou City (119.027–119.897° E, 32.232–33.397° N) is located in Jiangsu Province, China, with an altitude range of 0–81.71 m and an area of 6591.21 km^2^. Yangzhou City has a subtropical monsoon humid climate and an average annual temperature of 16.1 °C [17]. The main crops are rice, wheat, oilseed rape, vegetables, and various plantations.

### 2.2. Investigation Method

**Figure 1:**
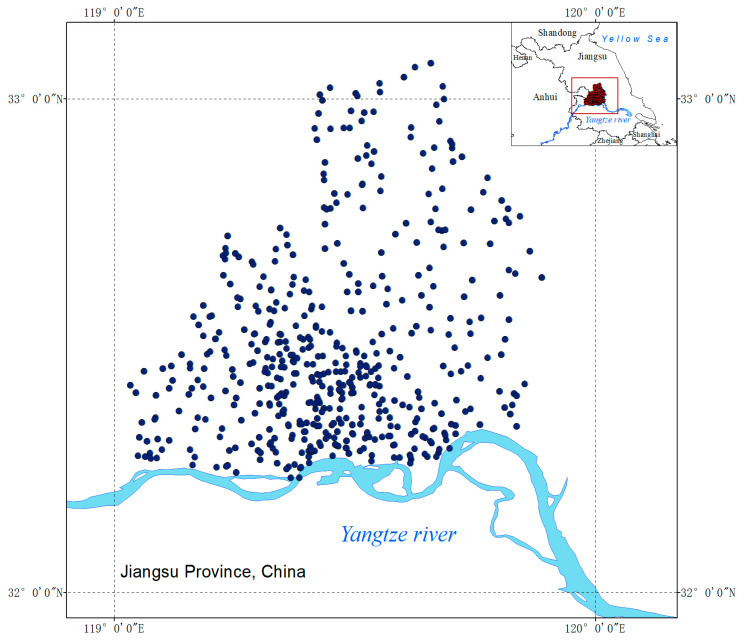
Distribution map of sites.

From September 2022 to July 2023, 666 random survey sites were set up in the agricultural planting areas in Yangzhou City (Figure 1). Each site surveyed an area of 3.4 hectares, which contained at least two different habitats, such as arable lands, irrigation canals and ditches, plantations, roads, or ponds. Field surveys were conducted during the lush growth period of weed communities in September and October 2022 and April, May, June, and July 2023. Each year, we drove around agricultural areas and selected sites randomly, and the interval between two adjacent sites was >3 km. At each site, the species of native weeds were recorded, and the occurrence dominance was estimated using a visual scoring method (Table 1), which was frequently used in weed community field surveys in China [18,19].

### 2.3. Traits of Weeds

We listed the life history traits of weeds studied here by referring to the Australian Weed Risk Assessment System (AWRA) [8] and the Agricultural Weed Assessment Calculator (AWAC) [20], as well as references on weed risk assessment conducted in China [5,6,21]. A total of 13 indicators were listed (Table 2), and each indicator was divided into two to four sorts of traits. The specific characteristics of each plant species and the categories to which they belong were determined mainly through field observations and by consulting the Flora of China website (https://www.iplant.cn/)(accessed on 5 May 2024).

### 2.4. Data Statistical Analysis

Comparisons of data among the three or four traits were subjected to one-way analysis of variance (ANOVA), using the SPSS 16.0 statistical package. The data were checked for normality and constant variance before analysis. The weed data were log-transformed before analysis. Nontransformed means for weed control were reported, with statistical interpretation based on transformed data. Treatment means were separated using the Fisher’s protected LSD test at *p* = 0.05. Independent sample t-tests were employed for comparisons with data between two sorts of traits, using SPSS 16.0. ArcGis 10.8 software was used to draw the distribution map of sites. The data presented are means ± SEs. To investigate the relationships among various traits of native weeds, we performed principal component analysis (PCA) based on the dominance values of traits collected from 666 sites. The analysis was conducted using the “vegan” package in R version 4.2.2 to evaluate the interactions among 13 different weed trait indicators [18,19].

## 3. Result

### 3.1. Weed Species Recorded in the Survey

**Figure 2:**
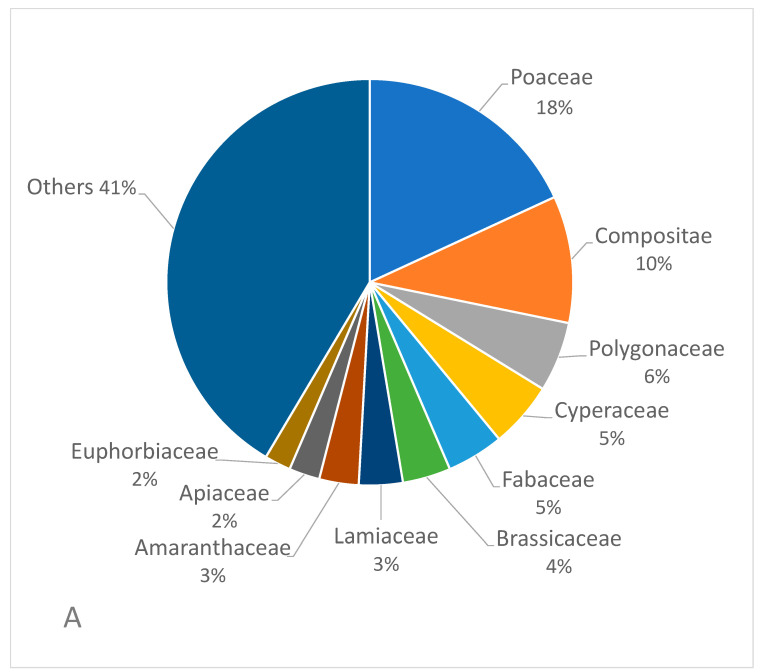

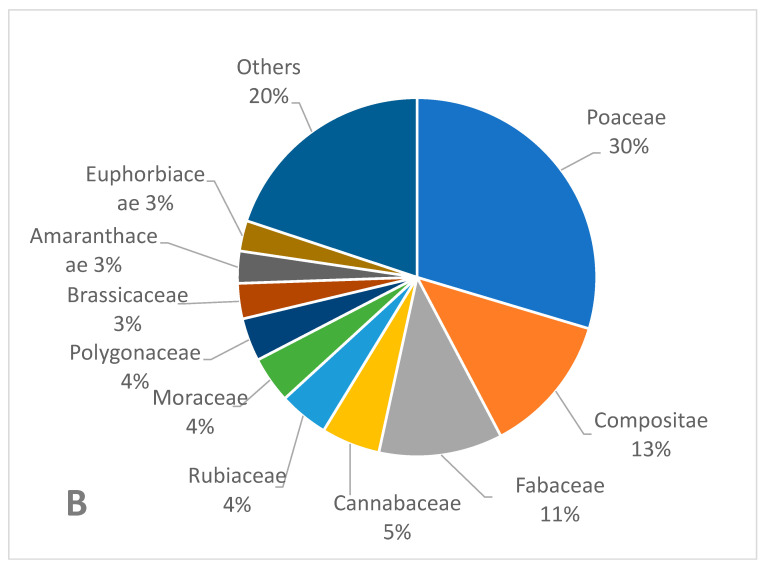
Species number (**A**) and dominance (**B**) of 287 native weed families.

A total of 287 native weed species were recorded (Table S1). These native weeds referred to 63 families, of which Poaceae contained the most species, with up to 52 species (Figure 2A), followed by Compositae (31), Polygonaceae (16), Cyperaceae (15), Fabaceae (13), Cruciferae (12), Lamiaceae (10), Amaranthaceae (9), Euphorbiaceae (7), Apiaceae (6), Cucurbitaceae (5), Lythraceae (5), Caryophyllaceae (5), Rosaceae (5), and Commelinaceae (5). Moreover, 26 families contained only one native weed species. A total of 192 genera were involved in these 287 species, among which *Echinochloa* contained most native weed species (9), followed by *Polygonum* (8). *Artemisia*, *Eragrostis*, *Cyperus*, *Rorippa,* and *Rumex* all contained five species, and *Setaria*, *Ranunculus*, *Solanum,* and *Vicia* all contained four species. There are nine genera containing three species, 27 genera containing two species, and 145 genera containing only one species.

In terms of the occurrence dominance (Figure 2B), the total dominance value of Poaceae weeds accounted for 30% of the total weed occurrence dominance value, followed by Compositae (13%), Fabaceae (11%), Cannabisaceae (5%), Rubiaceae (4%), Moraceae (4%), Polygonaceae (4%), Cruciferae (3%), Amaranthaceae (3%), and Euphorbiaceae (3%).

### 3.2. Plant Height

**Figure 3:**
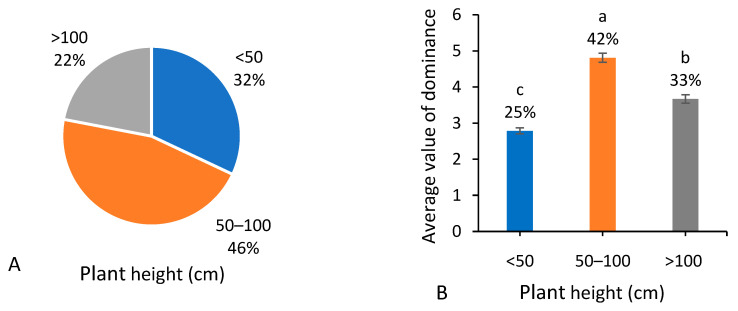
Number of native weed species (**A**) and average value of dominance among 666 field surveying sites (**B**) referring to different plant heights (cm). Note that each percentage value represents the proportion of each trait within the same group of indicators. In (**B**), different letters suggest significant differences at *p* < 0.05.

Among the 287 native weed species, 31% of species were <50 cm in plant height (low), 21% of species were >100 cm (tall), and 45% were 50–100 cm (moderate) in plant height (Figure 3A). In terms of the occurrence dominance (Figure 3B), the total dominance value of species with moderate plant height occupied (*p* < 0.05) the biggest proportion of the total weed occurrence dominance.

### 3.3. Life Type

**Figure 4:**
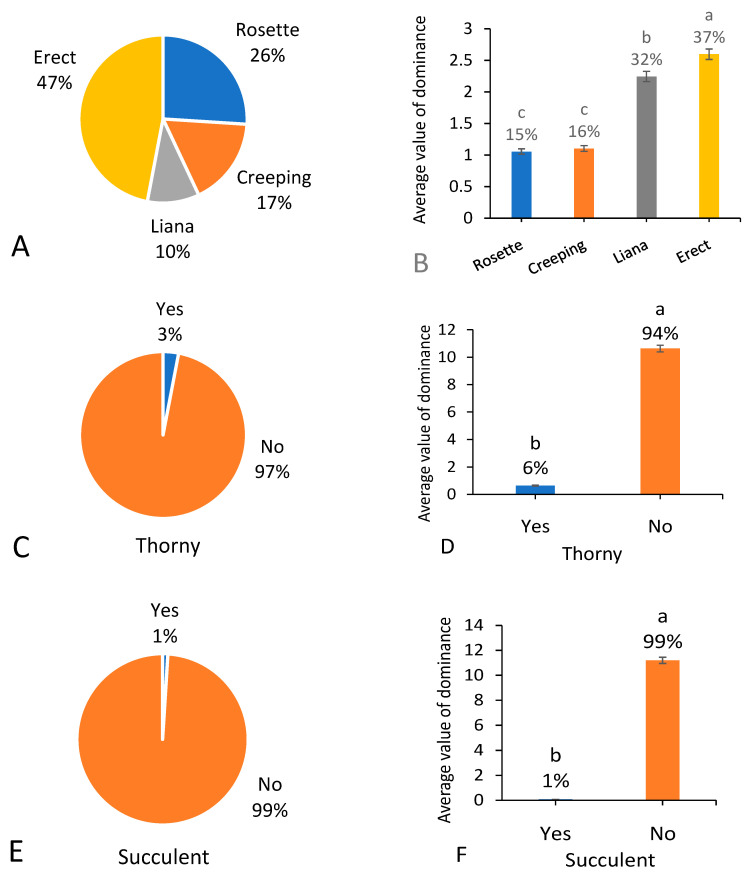
Number of native weed species (**A**,**C**,**E**) and average value of dominance among 666 field surveying sites (**B**,**D**,**F**) referring to different life types. Note that each percentage value represents the proportion of each trait within the same group of indicators. In (**B**,**D**,**F**) different letters suggest significant differences at *p* < 0.05.

According to the classification of life forms, 26% of the native weed species were rosette life forms (all the leaves being basal), 47% were erect, 18% were creeping plants, and 10% were lianas (Figure 4A). Moreover, 3% of the species had noticeable thorns or hooks (Figure 4C), and 1% were succulent plants (Figure 4E).

The total dominance value of native weed species with erect form occupied the biggest proportion of the total weed occurrence dominance, and the proportion decreased from 47% in species richness to 37% in occurrence dominance. Rosette plants also showed a decrease in the proportion of native weed occurrence dominance, compared to species richness. Meanwhile, the total dominance value of native liana weed species occupied 32% of the total native weed occurrence dominance, and the proportion was much higher than the proportion in species richness (Figure 4B). Besides, the proportions of occurrence dominance of native weed species with thorns and succulent plants were 6% and 1%, respectively (Figure 4D,F).

### 3.4. Drought Resistance

**Figure 5:**
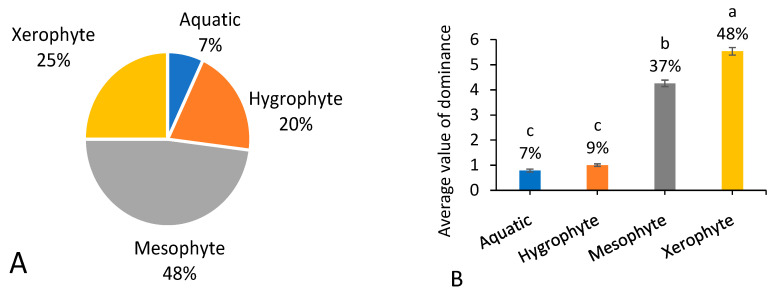
Number of native weed species (**A**) and average value of dominance among 666 field surveying sites (**B**) referring to different drought resistance. Note that each percentage value represents the proportion of each trait within the same group of indicators. In (**B**), different letters suggest significant differences at *p* < 0.05.

Among the 287 native weed species recorded, only 7% of species richness and occurrence dominance were aquatic plants (Figure 5A). *Phragmites australis* and *Leersia hexandra* can occur in aquatic and terrestrial habitats and thus were included in the list of aquatic and terrestrial weed species here. According to the adaptability to drought conditions, terrestrial weed species were further divided into three categories: hygrophytes (not tolerant to drought stress), xerophytes (drought tolerance), and mesophytes (with drought tolerance between hygrophytes and xerophytes). Mesophytes occupied the biggest proportion of native weed species in species richness (48%) but not in occurrence dominance (37%). Xerophytes showed an increase in the proportion of native weed species from species richness (25%) to occurrence dominance (48%). In comparison, hygrophytes showed a decrease in the proportion of native weed species from species richness (20%) to occurrence dominance (9%) (Figure 5B).

### 3.5. Shade Tolerance

**Figure 6:**
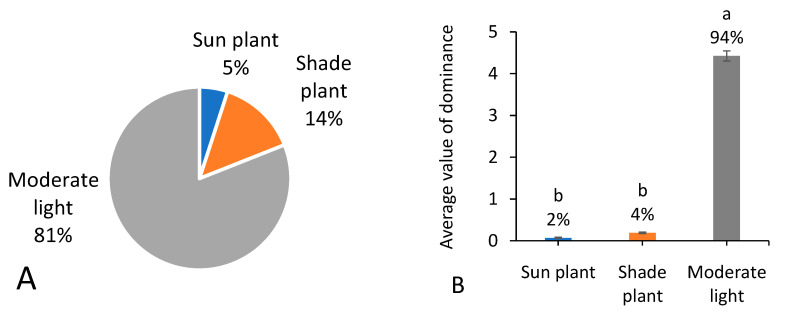
Number of native weed species (**A**) and average value of dominance among 666 field surveying sites (**B**) referring to different shade tolerance. Note that each percentage value represents the proportion of each trait within the same group of indicators. In (**B**), different letters suggest significant differences at *p* < 0.05.

According to the abilities in shade tolerance, weed species were divided into three categories: shade plants (adapted to under-canopy conditions), sun plants (plants that grow and develop vigorously in strong light environments and grow poorly under shade and weak light conditions), and moderate plants (plants holding shade tolerance between sun plants and shade plants). Among the 287 recorded native weed species (Figure 6A,B), a majority were moderate plants in both species richness (81%) and occurrence dominance (94%).

### 3.6. Life History Trait

**Figure 7:**
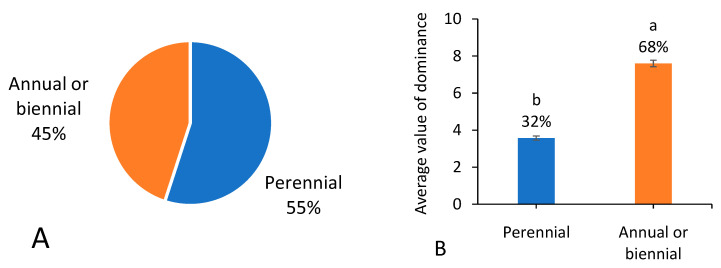
Number of native weed species (**A**) and average value of dominance among 666 field surveying sites (**B**) referring to different life history traits. Note that each percentage value represents the proportion of each trait within the same group of indicators. In (**B**), different letters suggest significant differences at *p* < 0.05.

Among the 287 native weed species, 55% were perennials, while the total occurrence dominance of perennials was significantly much lower than those of annual or biennial weeds (Figure 7A,B).

### 3.7. Reproductive Characteristics

**Figure 8:**
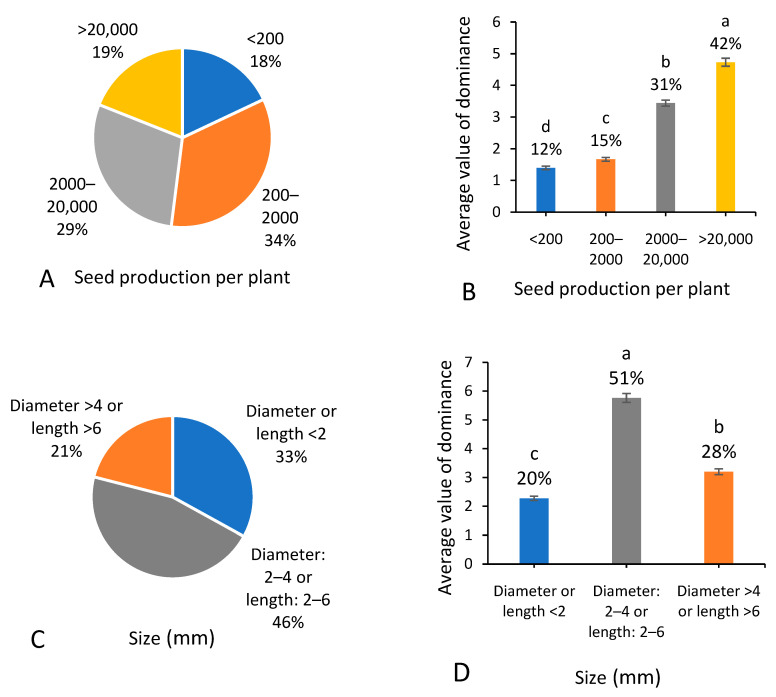

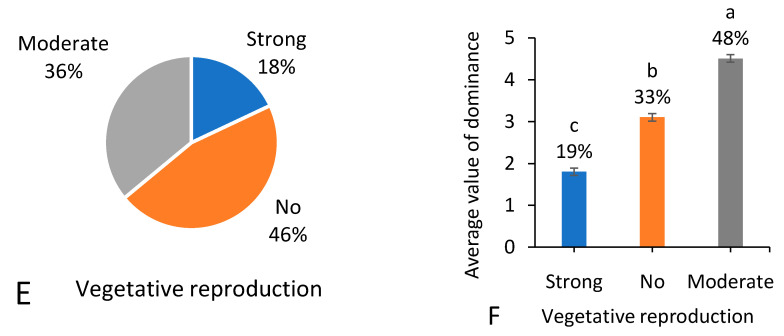
Number of native weed species (**A**,**C**,**E**) and average value of dominance among 666 field surveying sites (**B**,**D**,**F**) referring to different reproductive characteristics. Note that each percentage value represents the proportion of each trait within the same group of indicators. In (**B**,**D**,**F**), different letters suggest significant differences at *p* < 0.05.

Among the 287 native weed species recorded, 18% species held low fertility (seed production per plant usually <200) (Figure 8A), 34% held moderate fertility (seed production per plant usually between 200 and 2000), 29% held high fertility (seed production per plant usually between 2001 and 20,000), and 19% held very high fertility (seed production per plant usually >20,000). Regarding occurrence dominance (Figure 8B), native weed species holding very high fertility increased in proportion out of the total native weeds, from 19% in species richness to 42% in occurrence dominance. Moreover, the native weed species holding moderate fertility showed a big decrease in the proportion, from 34% in species richness to 15% in occurrence dominance.

Regarding the size (Figure 8C), 33% out of the total 287 native weed species were tiny (<2 mm in diameter or length), 46% were moderate (2–4 mm in diameter or 2–6 mm in length), and 21% were bigger (>4 mm in diameter or >6 mm in length). Native weed species with moderate seeds occupied more than half (51%) of the total occurrence dominance of the overall native weed species, and native weed species with bigger seeds showed an increase from 21% in species richness to 28% in occurrence dominance (Figure 8D).

Regarding the ability of natural vegetative reproduction (Figure 8E), weed species were divided into three categories: low (plants usually do not reproduce and spread by leaves, roots, or stems), strong (plants readily reproduce and spread by leaves, roots, or stems), and moderate (plants hold the ability of vegetative reproduction but usually reproduce and spread by seeds). Among the 287 native weed species, 46% held a low ability, 36% held a moderate ability, and 18% held a strong ability of vegetative reproduction. In terms of occurrence dominance, species holding a moderate ability of vegetative reproduction occupied the highest proportion out of the total occurrence dominance of the overall native weed species (Figure 8F).

### 3.8. Appendages of Fruits

**Figure 9:**
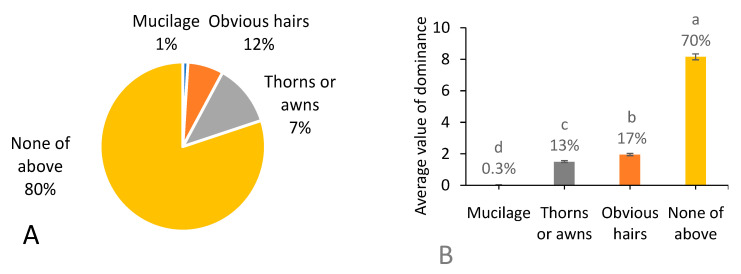
Number of native weed species (**A**) and average value of dominance among 666 field surveying sites (**B**) referring to different appendages of fruits. Note that each percentage value represents the proportion of each trait within the same group of indicators. In (**B**), different letters suggest significant differences at *p* < 0.05.

Among the 287 native weed species (Figure 9A), 1% held mucilage on the fruit surface, fruits of 12% of the species showed obvious hairs, fruits of 7% of the species showed thorns or awns, and 80% of the species did not show mucilage, obvious hairs, thorns or awns. With regards to occurrence dominance, the proportions of native weeds with fruits holding appendages, including obvious hairs, thorns, or awns, occupied increasing proportions compared with the proportions in species richness. Whereas 70% of the occurrence dominance was still occupied by native weeds with fruits without the above appendages (Figure 9B).

### 3.9. Herbicide Resistance and Weediness

**Figure 10:**
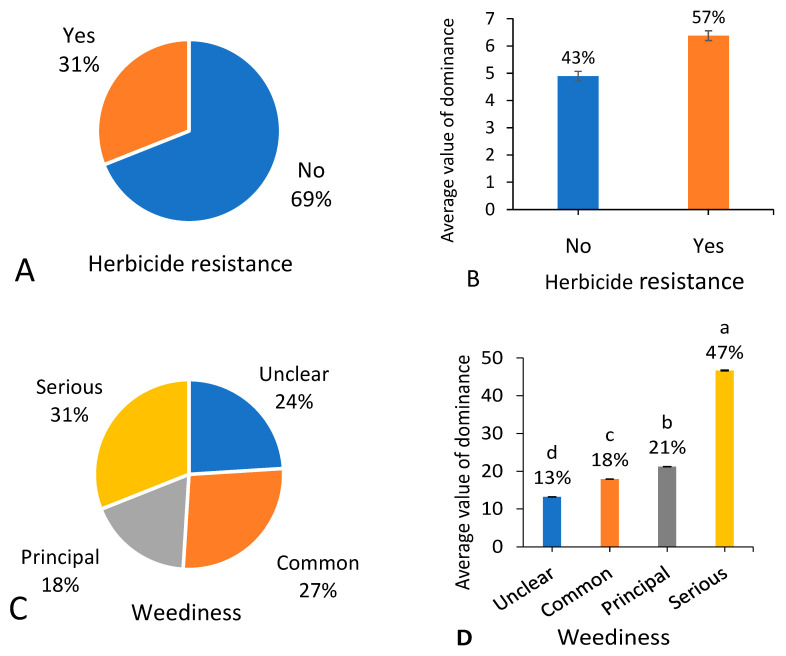
Number of native weed species (**A**,**C**) and average value of dominance among 666 field surveying sites (**B**,**D**) referring to different herbicide resistance potential. Note that each percentage value represents the proportion of each trait within the same group of indicators. In (**B**,**D**), different letters suggest significant differences at *p* < 0.05.

Among the 287 native weed species, 31% were found to have herbicide-resistant populations or biotypes worldwide (Figure 10A), which occupied 57% occurrence dominance out of the total native weeds (Figure 10B). According to *A Geographical Atlas of World Weeds* [23], 31% out of the total 287 native weed species were recorded as a serious weed in at least one country or area (Figure 10C), 18% were recorded as a principal weed in at least one country or area but not recorded as a serious weed, 27% were recorded as a common weed in at least one country or area but not recorded as a serious or principal weed, and the remaining 24% were listed as holding unclear weediness. Serious weeds showed a significantly higher proportion (47%) in occurrence dominance among the overall native weeds (Figure 10D). Moreover, the total occurrence dominance values of weed groups increased with increasing weediness.

### 3.10. PCA of Weed Traits

**Figure 11:**
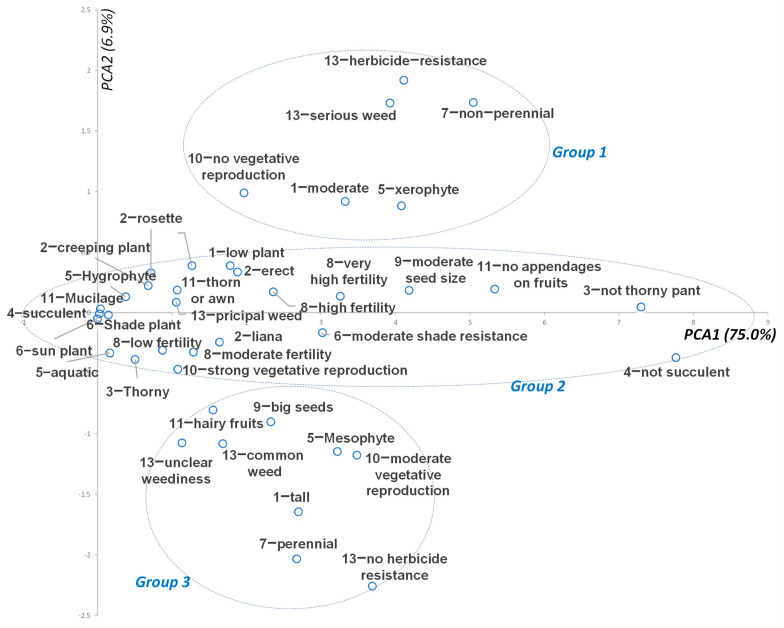
Principal component analysis (PCA) showing the 13 weed trait indicators. Note that the codes of indicators are the same as in Table 2.

The results extracted from the PCA show that 81.8% of the total variance of the data obtained could be explained using the first two principal components (Figure 11). According to the PCA results, the traits of native weeds could be organized into 3 groups. Group 1 is composed of serious weeds recorded in “*A Geographical Atlas of World Weeds*”, herbicide resistance, non-perennial, no vegetative reproduction, moderate plant height (50–100 cm), and xerophyte referring to drought tolerance. Group 2 is composed of principal weed and various weed traits. Group 3 is composed of common weeds and plants with unclear weediness, hairy fruits, big seeds, perennials, tall plants, moderate vegetative reproduction, and mesophytes referring to drought resistance.

## 4. Discussion

### 4.1. Native Weed Diversity in Agricultural Areas

Based on the surveys of hundreds of field sites in agricultural areas, this is the first study to reveal the diversity of native weed flora in a city in China. Weed Flora of China [24] listed 1454 weed species, and Alien Invasive Flora of China [25] listed 403 alien invasive plant species in China. Thus, a list of 1360 native weed species was generated. The surveyed city, Yangzhou, represents about 0.07% of the land area in mainland China, while the number of native weed species represents about 21% of the total native weed species in China. Hence, as a city in eastern China, Yangzhou has a rich diversity of weed flora species. Meanwhile, eastern China is also one of the hottest areas suffering alien plant invasions, and a relatively effective risk assessment system is urgently needed. The conclusions from this study can provide reference and suggestions for the establishment of such a risk assessment system in the future.

Poaceae is a species-rich plant family consisting of about 10,000 species (commonly called grasses), including the most economically important plants [26]. It has been suggested that the genomes of Poaceae have evolved at an elevated rate due to the selection imposed by changing environmental conditions and more recent breeding efforts [27,28]. Grasses usually represent the most troublesome weed groups in various agricultural areas, particularly in cereal crops. For example, many species of grassy weeds have become the dominant malignant weeds in wheat, rice, and corn fields worldwide [29,30]. In our survey, grassy weeds also occupied the biggest proportions in both species richness (18%) and occurrence dominance (30%) out of the total native weed species. Hence, alien plant species belonging to Poaceae should be highlighted with higher scores in risk-assessing systems for agricultural areas.

Compositae is one of the largest and most diverse plant families in the world, with more than 26,000 species, accounting for about 7% of the existing flowering plants [31]. Compositae is distributed across all regions of the world regions except for Antarctica [32,33] and exists in almost all habitats [34]. In addition, many Compositae plants are used as medicine and/or food plants [35]. Regarding alien plant invasions, Compositae plants account for 10% of the world’s naturalized alien plants, which is the biggest plant family [36]. In this study, Compositae was second to Poaceae in both species richness and occurrence dominance of native weeds. Hence, alien plant species belonging to Compositae should also be highlighted with higher scores in risk-assessing systems for agricultural areas. Moreover, Fabaceae should also be highlighted in risk-assessing systems for agricultural areas. Fabaceae is the third largest plant family after Compositae and Orchidaceae in the world, with about 19,500 species [37,38,39]. Fabaceae are usually rich in protein and are the main dietary source in many countries and regions, and many Fabaceae plant species are crops or medicine plants [39,40]. In the list of alien invasive plant species, Fabaceae account for 15% in South Asia [41], 7% in Europe [42], and 11% in China [43]. In addition, among the 287 native weed species, Cyperaceae, Cruciferae, Labiatae, and Polygonaceae also accounted for a higher proportion. It is recommended to pay attention to these plant families in risk-assessing systems for agricultural areas. For example, Cyperaceae is the second largest weed group after Poaceae weeds [44].

### 4.2. Traits Closely Related to Weediness in Agricultural Areas

To date, several countries or areas have established alien plant invasion risk assessment systems, such as Australia, the United States, New Zealand, Central Europe, and China [5,9,11,20]. In China, there were studies that established alien plant invasion risk assessment systems on a regional scale, for example, for Xiamen City [21] or Ningbo City [45]. These risk assessment systems were mainly based on assignments of life history traits of plants. Together, our results suggested significant differences in relationships between different traits and occurrence dominance in native weed species in agricultural areas.

Weeds live in habitats with high levels of human disturbances, particularly in agricultural areas, where weed management practices are frequent and periodic. Weeds adopting the R strategy [46] are more adapted to agricultural environments, which learn to generate many seeds in a shorter life span, such as annuals or biennials with high fertility [1]. Meanwhile, perennials with high fertility and vegetative reproduction could also be highly adapted to agricultural environments, as vegetative reproduction facilitates the colonization and diffusion of weeds [47]. In our study, native weeds holding a shorter life span (annuals of biennials) were more dominant than perennials; native weeds holding moderate ability of vegetative reproduction were more dominant than those holding high ability of vegetative reproduction; and native weeds holding high fertility showed big competitive advantages reflected by occurrence dominance. Interestingly, native weeds with moderate seed sizes showed big competitive advantages compared with those with big seeds or those with tiny seeds. Agricultural environments are usually characterized by rich resources for plant growth. Quick emergence from soil layers and fast growth could be important to occupy great advantages in these habitats. The nutrition contained in seeds is almost the only source of seedling emergence and very early growth. Thus, the moderate seed sizes outweigh the tiny seeds. Moreover, given the limited nutrition in a plant, large seeds often result in much lower seed production. Considering that weeds recorded in agricultural areas tend to be non-perennial with a plant height of < 1 m, moderate seed sizes also outweigh large seeds.

In agricultural areas usually holding rich resources, taller weed species might quickly occupy preferable niches and form dominant communities [48]. Whereas tall weeds are also more likely to attract attention for weed management. Weeds with moderate plant height showed advantages compared with tall or low weeds. Moreover, tall plants commonly need longer periods for growing, which may be frequently disturbed by weed management practices. Erect plants were most common among native weed species in the agricultural area surveyed. In addition, the upright plant surface is relatively less exposed to herbicides, and thus, many troublesome weed species holding serious herbicide resistance are erect plants [49]. Moreover, lianas showed competitive advantages in occurrence dominance for achieving a larger area of coverage with smaller biomass. Succulent plants usually grow slower, have a higher ability to adapt to drought stress, and are not advantageous in capricious agricultural environments with rich resources. Although studies have shown that plants with thorns are more likely to become invasive alien plants [5], plants with obvious thorns are frequently controlled in agricultural areas due to their potential to cause stab wounds.

Kuester et al. [50] reported that hygrophytes are more likely to become invasive plants. At the same time, our survey suggested that xerophytes and mesophytes accounted for 73% of the species richness and 85% of the occurrence dominance among the overall native weeds. In eastern China, most crops are highland crops, and highlands host most native weed species. In addition, the weed diversity in paddy fields in eastern China is relatively lower, among which many weed species occurring on paddy fields are mesophytes, such as *Echinochloa* spp., *Leptochloa chinensis*, and *Cyperus iria* [51].

*A Geographical Atlas of World Weeds* [22] provides a comprehensive international review of approximately 8000 species of weeds, compiling geographic distributions for the countries or areas where a species is known to occur as a weed of serious, principal, common, or unknown seriousness (ranking provided by a weed scientist from that country), or where it is present in the general flora but not known to act as a weed. Our survey suggested that the occurrence dominance of native weeds increased with increasing weediness of native weed groups. Therefore, this book could still be an important reference for risk-assessing systems for agricultural areas.

Moreover, the PCA indicated that serious weeds were more likely to exhibit traits such as evolving herbicide resistance, herbicide resistance, non-perennial growth, lack of vegetative reproduction, moderate plant height, and xerophytic characteristics associated with drought tolerance. Therefore, these traits should be emphasized when assessing the invasive risks of introducing plants into agricultural areas.

Applying chemical herbicides is the most important weed management strategy in agricultural areas [52,53]. Many weed species have evolved resistance to various herbicides and caused serious damage to agriculture worldwide [54]. Weed species that have evolved herbicide resistance are frequently characterized by high fertility to produce a large number of offspring with high genetic diversity for herbicide selection, or they are highly adapted to certain or many kinds of agricultural habitats, or they have a high ability for regrowth or quick seedling growth to escape herbicide applications. Thus, herbicide-resistant weed species might also be very troublesome and evolve herbicide resistance in introduced regions. On the other hand, herbicide-resistant populations or biotypes might be directly introduced into new regions by various means [55]. Therefore, information on herbicide resistance elsewhere could also be important for risk-assessing systems for agricultural areas.

## 5. Conclusions

This study underscored the high diversity of native weeds and their adaptive characteristics in eastern China and highlighted the predominance of Poaceae and Compositae in both species richness and occurrence dominance. Together, our results indicated that native weeds with high fertility, moderate seed sizes, shorter life spans, and intermediate vegetative reproduction abilities tend to exhibit greater dominance, reflecting their adaptive strategies in agricultural environments. Moreover, traits such as plant height, growth form, and herbicide resistance play crucial roles in weed competitiveness and management challenges. The observed patterns and traits of the native weeds offer valuable information for understanding their ecological roles and improving weed management practices in agricultural landscapes. The insights are pivotal for refining risk assessment systems for plant invasions in agricultural areas and developing targeted management strategies.

## Figures and Tables

**Table 1:** Visual scoring method for weed dominance value in crop fields.

Code	Maximum Height of Weeds in the Field		
	>80 cm ^a^	20 cm–80 cm ^b^	<20 cm ^c^
0.1	1–3 stems or total coverage <0.1%	<10 stems or total coverage <1%	<15 stems or total coverage <2%
0.5	4–10 stems or total coverage 0.2–0.9%	11–15 stems or total coverage 1–2%	16–30 stems or total coverage 3–5%
1	11–15 stems or total coverage 1–2%	16–30 stems or total coverage 3–5%	31–60 stems or total coverage 6–10%
2	16–30 stems or total coverage 3–5%	31–60 stems or total coverage 6–10%	61–100 stems or total coverage 11–25%
3	31–60 stems or total coverage 6–10%	61–100 stems or total coverage 11–25%	101–200 stems or total coverage 25–50%
4	61–100 stems or total coverage 11–25%	101–200 stems or total coverage 25–50%	201–500 stems or total coverage 50–90%
5	>100 stems or total coverage >25%	>200 stems or total coverage >50%	>500 stems or total coverage >90%

^a^: Near or above the crop. ^b^: In the middle of the crop canopy. ^c^: At lower heights within the crop.

**Table 2:** Weed trait indicators.

Code	Indicator	Traits
1	Plant height	>100 cm (tall), 50–100 cm (moderate), or <50 cm (low)
2	Life type	Rosette, erect, creeping, or liana
3	Thorns or hooks	Yes or no
4	Succulent	Yes or no
5	Drought resistance	Aquatic, hygrophyte, mesophyte, or xerophyte
6	Shade tolerance	Shade plant, moderate, sun plant
7	Life span	Annual or biennial or perennial
8	Seed production per plant	<200 (low fertility), 200–2000 (moderate fertility), 2000–20,000 (high fertility), or >20,000 (very high fertility)
9	Size (mm)	Diameter or length < 2 (tiny), 2–4 in diameter or 2–6 in length (moderate), or diameter > 4 or length > 6 (big)
10	Vegetative reproduction	No, moderate, or strong
11	Appendages of fruits	Mucilage, obvious hairs, thorns or awns, or none of the above
12	Herbicide resistance ^a^	Yes or no
13	Weediness ^b^	Unclear, common, principal, or serious

^a^: According to HRAC (weedscience.org/Home.aspx) (accessed on 20 May 2024) researching reports in Web of Science (webofscience.clarivate.cn/wos/alldb/basic-search) (accessed on 20 May 2024), and CNKI (www.cnki.net/) (accessed on 20 May 2024). ^b^: According to *A Geographical Atlas of World Weeds* (Holm et al. 1979) [22].

## Data Availability

The original contributions presented in the study are included in the article and Supplementary Material.

## Supplementary Materials

The following supporting information can be downloaded at: https://www.mdpi.com/article/10.3390/biology13090704/s1, Table S1: 287 species of native weeds and their families were investigated.
