# Supplementary material for: Diversity and Life History Traits of Native Weed Communities in Agricultural Areas: A Case Study in Eastern China
Source: Biology (Basel). 2024 Sep 7;13(9):704. doi: 10.3390/biology13090704 (PMC11429208; doi:10.3390/biology13090704)
Supplement: Supplementary file 1 [file biology-13-00704-s001.zip › biology-3153818-supplementary.pdf]

Table S1. 287 species of native weeds and their families were investigated.

Code	Family	Species
1	Acanthaceae	*Justicia procumbens* L.
2	Acoraceae	*Acorus calamus* L.
3	Alismataceae	*Sagittaria pygmaea* Miq.
4		*Sagittaria trifolia* subsp. *leucopetala* (Miq.) Q. F. Wang
5	Amaranthaceae	*Achyranthes aspera* L.
6		*Achyranthes bidentata* Blume
7		*Alternanthera sessilis* (L.) R. Br. ex DC.
8		*Bassia scoparia* (L.) A. J. Scott
9		*Chenopodium album* L.
10		*Chenopodium ficifolium* Sm.
11		*Chenopodium karoi* (Murr) Aellen
12		*Oxybasis glauca* (L.) S. Fuentes, Uotila & Borsch
13		*Celosia argentea* L.
14	Amaryllidaceae	*Allium macrostemon* Bunge
15	Apiaceae	*Centella asiatica* (L.) Urb.
16		*Cnidium monnieri* (L.) Spreng.
17		*Daucus carota* L.
18		*Oenanthe javanica* (Blume) DC.
19		*Torilis japonica* (Houtt.) DC.
20		*Torilis scabra* (Thunb.) DC.
21	Apocynaceae	*Cynanchum rostellatum* (Turcz.) Liede & Khanum
22		*Trachelospermum jasminoides* (Lindl.) Lem.
23	Araceae	*Lemna minor* L.
24		*Spirodela polyrhiza* (L.) Schleid.
25		*Pinellia ternata* (Thunb.) Ten. ex Breitenb.
26	Boraginaceae	*Bothriospermum zeylanicum* (J. Jacq.) Druce
27		*Bothriospermum chinense* Bunge
28		*Lithospermum arvense* L.
29		*Trigonotis peduncularis* (Trevis.) Benth. ex Baker & S. Moore
30	Brassicaceae	*Brassica juncea* (L.) Czern.
31		*Capsella bursa-pastoris* (L.) Medik.
32		*Cardamine flexuosa* With.
33		*Cardamine occulta* Hornem.
34		*Descurainia sophia* (L.) Webb ex Prantl
35		*Orychophragmus violaceus* (L.) O. E. Schulz
36		*Rorippa cantoniensis* (Lour.) Ohwi
37		*Rorippa dubia* (Pers.) Hara
38		*Rorippa indica* (L.) Hiern
39		*Rorippa palustris* (L.) Besser
40		*Thlaspi arvense* L.
41	Campanulaceae	*Lobelia chinensis* Lour.
42	Cannabaceae	*Humulus scandens* (Lour.) Merr.
43	Caprifoliaceae	*Lonicera japonica* Thunb.
44	Caryophyllaceae	*Arenaria serpyllifolia* L.

45		*Gypsophila vaccaria* Sm.
46		*Sagina japonica* (Sw.) Ohwi
47		*Stellaria aquatica* (L.) Scop.
48		*Stellaria media* (L.) Vill.
49	Celastraceae	*Euonymus fortunei* (Turcz.) Hand.-Mazz.
50	Commelinaceae	*Commelina benghalensis* L.
51		*Commelina communis* L.
52		*Commelina diffusa* Burm. f.
53		*Murdannia nudiflora* (L.) Brenan
54		*Murdannia triquetra* (Wall.) Bruckn.
55	Compositae	*Artemisia annua* L.
56		*Artemisia argyi* H. Lév. & Vaniot
57		*Artemisia indica* Willd.
58		*Artemisia integrifolia* L.
59		*Artemisia mongolica* (Fisch. ex Besser) Nakai
60		*Artemisia scoparia* Waldst. & Kit.
61		*Aster indicus* L.
62		*Aster pekinensis* (Hance) F. H. Chen
63		*Carduus nutans* L.
64		*Carpesium abrotanoides* L.
65		*Centipeda minima* (L.) A. Braun & Asch.
66		*Chrysanthemum indicum* L.
67		*Cirsium arvense* var. *integrifolium* Wimm. & Grab.
68		*Crepidiastrum sonchifolium* (Bunge) Pak & Kawano
69		*Eclipta prostrata* (L.) L.
70		*Hemisteptia lyrata* (Bunge) Fisch. & C. A. Mey.
71		*Inula japonica* Thunb.
72		*Ixeridium dentatum* (Thunb.) Tzvelev
73		*Ixeris polycephala* Cass. ex DC.
74		*Lactuca formosana* Maxim.
75		*Lactuca indica* L.
76		*Lapsanastrum apogonoides* (Maxim.) Pak & K. Bremer
77		*Pseudognaphalium affine* (D. Don) Anderb.
78		*Sigesbeckia orientalis* L.
79		*Sonchus oleraceus* L.
80		*Sonchus wightianus* DC.
81		*Taraxacum mongolicum* Hand.-Mazz.
82		*Youngia erythrocarpa* (Vaniot) Babc. & Stebbins
83		*Youngia japonica* (L.) DC.
84	Convolvulaceae	*Calystegia hederacea* Wall.
85		*Cuscuta australis* R. Br.
86		*Dichondra micrantha* Urb.
87	Crassulaceae	*Sedum bulbiferum* Makino
88	Cucurbitaceae	*Actinostemma tenerum* Griff.
89		*Cucumis melo* var. *agrestis* Naudin
90		*Thladiantha nudiflora* Hemsl. ex Forbes & Hemsl.
91		*Trichosanthes kirilowii* Maxim.

92		*Zehneria japonica* (Thunb.) H. Y. Liu
93	Cyperaceae	*Carex breviculmis* R. Br.
94		*Cyperus compressus* L.
95		*Cyperus difformis* L.
96		*Cyperus iria* L.
97		*Cyperus microiria* Steud.
98		*Cyperus rotundus* L.
99		*Cyperus serotinus* Rottb.
100		*Eleocharis dulcis* (Burm. f.) Trin. ex Hensch.
101		*Fimbristylis aestivalis* (Retz.) Vahl
102		*Fimbristylis littoralis* Gaudich.
103		*Fimbristylis rigidula* Nees
104		*Kyllinga polyphylla* Kunth
105		*Pycreus flavidus* (Retz.) T. Koyama
106		*Pycreus sanguinolentus* (Vahl) Nees ex C. B. Clarke
107		*Schoenoplectus triqueter* (L.) Palla
108	Dioscoreaceae	*Dioscorea polystachya* Turcz.
109	Equisetaceae	*Equisetum arvense* L.
110		*Equisetum hyemale* L.
111	Euphorbiaceae	*Acalypha australis* L.
112		*Acalypha brachystachya* Hornem.
113		*Euphorbia helioscopia* L.
114		*Euphorbia makinoi* Hayata
115		*Euphorbia thymifolia* L.
116		*Euphorbia humifusa* Willd. ex Schltdl.
117		*Euphorbia lathyris* L.
118	Fabaceae	*Aeschynomene indica* L.
119		*Glycine soja* auct. non Siebold & Zucc. : Baker
120		*Kummerowia stipulacea* (Maxim.) Makino
121		*Kummerowia striata* (Thunb.) Schindl.
122		*Lespedeza bicolor* Turcz.
123		*Medicago lupulina* L.
124		*Pueraria edulis* Pamp.
125		*Vicia cracca* L.
126		*Vicia hirsuta* (L.) Gray
127		*Vicia sativa* L.
128		*Vicia tetrasperma* (L.) Moench
129		*Medicago minima* (L.) Lam.
130		*Senna tora* (L.) Roxb.
131	Haloragaceae	*Myriophyllum verticillatum* L.
132	Hydrocharitaceae	*Hydrilla verticillata* (L. f.) Royle
133		*Hydrocharis dubia* (Blume) Backer
134		*Najas minor* All.
135		*Vallisneria natans* (Lour.) H. Hara
136	Juncaceae	*Juncus effusus* L.
137	Lamiaceae	*Glechoma longituba* (Nakai) Kupr.
138		*Lamium amplexicaule* L.

139		*Leonurus japonicus* Houtt.
140		*Lycopus lucidus* Turcz. ex Benth.
141		*Mentha canadensis* L.
142		*Mosla scabra* (Thunb.) C. Y. Wu & H. W. Li
143		*Perilla frutescens* (L.) Britton
144		*Prunella vulgaris* L.
145		*Salvia plebeia* R. Br.
146		*Stachys japonica* Miq.
147	Linderniaceae	Lindernia *antipoda* (L.) Alston
148		Lindernia *crustacea* (L.) F. Muell.
149		Lindernia *procumbens* (Krock.) Borbás
150	Lygodiaceae	*Lygodium japonicum* (Thunb.) Sw.
151	Lythraceae	*Ammannia auriculata* Willd.
152		*Ammannia baccifera* L.
153		*Ammannia multiflora* Roxb.
154		*Lythrum salicaria* L.
155		*Rotala indica* (Willd.) Koehne
156	Malvaceae	*Abelmoschus manihot* (L.) Medik.
157		*Corchorus aestuans* L.
158		*Melochia corchorifolia* L.
159		*Sida acuta* Burm. F.
160	Marsileaceae	*Marsilea quadrifolia* L.
161	Mazaceae	*Mazus pumilus* (Burm. f.) Steenis
162	Meliaceae	*Melia azedarach* L.
163	Menispermaceae	*Cocculus orbiculatus* (L.) DC.
164	Molluginaceae	*Trigastrotheca stricta* (L.) Thulin
165	Moraceae	*Broussonetia papyrifera* (L.) L'Hér. ex Vent.
166		*Morus alba* L.
167		*Maclura tricuspidata* Carrière
168	Onagraceae	*Epilobium hirsutum* L.
169		*Ludwigia hyssopifolia* (G. Don) Exell
170		*Ludwigia adscendens* (L.) Hara
171		*Ludwigia prostrata* Roxb.
172	Oxalidaceae	*Oxalis corniculata* L.
173	Papaveraceae	*Corydalis edulis* Maxim.
174		*Papaver rhoeas* L.
175	Phyllanthaceae	*Phyllanthus urinaria* L.
176		*Phyllanthus ussuriensis* Rupr. & Maxim.
177	Plantaginaceae	*Limnophila sessiliflora* (Vahl) Blume
178		*Plantago asiatica* L.
179		*Plantago major* L.
180		*Veronica anagallis-aquatica* L.
181	Poaceae	*Alopecurus aequalis* Sobol.
182		*Alopecurus japonicus* Steud.
183		*Arthraxon hispidus* (Thunb.) Makino
184		*Arundo donax* L.
185		*Beckmannia syzigachne* (Steud.) Fernald

186	*Bothriochloa ischaemum* (L.) Keng
187	*Bromus japonicus* Thunb.
188	*Coix lacryma-jobi* L.
189	*Cynodon dactylon* (L.) Persoon
190	*Digitaria ischaemum* (Schreb.) Muhl.
191	*Digitaria radicosa* (J. Presl) Miq.
192	*Digitaria sanguinalis* (L.) Scop.
193	*Echinochloa caudata* Roshev.
194	*Echinochloa colona* (L.) Link
195	*Echinochloa crus-galli* (L.) P. Beauv.
196	*Echinochloa crus-galli* var. *mitis* (Pursh) Petermann
197	*Echinochloa crus-galli* var. *austrojaponensis* Ohwi
198	*Echinochloa hispidula* (Retz.) Nees.
199	*Echinochloa crus-galli* var. *zelayensis* (Kunth) Hitchcock
200	*Echinochloa cruspavonis* (H. B. K.) Schult.
201	*Echinochloa glabrescens* Munro ex Hook. f.
202	*Eleusine indica* (L.) Gaertn.
203	*Elymus kamoji* (Ohwi) S. L. Chen
204	*Eragrostis autumnalis* Keng
205	*Eragrostis ferruginea* (Thunb.) P. Beauv.
206	*Eragrostis japonica* (Thunb.) Trin.
207	*Eragrostis minor* Host
208	*Eragrostis pilosa* (L.) P. Beauv.
209	*Eragrostis tenella* (L.) P. Beauv. ex Roemer & Schult.
210	*Leersia hexandra* Sw.
211	*Leptochloa chinensis* (L.) Nees
212	*Leptochloa panicea* (Retz.) Ohwi
213	*Microstegium vimineum* (Trin.) A. Camus
214	*Miscanthus floridulus* (Labill.) Warburg ex K. Schumann
215	*Miscanthus sacchariflorus* (Maxim.) Benth. & Hook. f. ex Franch.
216	*Oryza sativa* f. spontanea
217	*Eriochloa villosa* (Thunb.) Kunth
218	*Panicum bisulcatum* Thunb.
219	*Paspalum thunbergii* Kunth ex Steud.
220	*Pennisetum alopecuroides* (L.) Spreng.
221	*Phleum paniculatum* Huds.
222	*Phragmites australis* (Cav.) Trin. ex Steud.
223	*Poa acroleuca* Steud.
224	*Poa annua* L.
225	*Polypogon fugax* Nees ex Steud.
226	*Pseudosclerochloa kengiana* (Ohwi) Tzvelev
227	*Setaria faberi* R. A. W. Herrmann
228	*Setaria forbesiana* (Nees ex Steud.) Hook. f.
229	*Setaria pumila* (Poir.) Roem. & Schult.
230	*Sporobolus fertilis* (Steud.) Clayton
231	*Zizania latifolia* (Griseb.) Turcz. ex Stapf
232	*Setaria viridis* (L.) P. Beauv.

233	Polygonaceae	*Persicaria hydropiper* (L.) Spach
234		*Persicaria lapathifolia* var. *salicifolia* (Sibth.) Miyabe
235		*Persicaria lapathifolia* (L.) Delarbre
236		*Persicaria longiseta* (Bruijn) Moldenke
237		*Persicaria maculosa* Gray
238		*Persicaria orientalis* (L.) Spach
239		*Persicaria perfoliata* (L.) H. Gross
240		*Persicaria posumbu* (Buch.-Ham. ex D. Don) H. Gross
241		*Polygonum aviculare* L.
242		*Polygonum plebeium* R. Br.
243		*Reynoutria japonica* Houtt.
244		*Rumex crispus* L.
245		*Rumex dentatus* L.
246		*Rumex japonicus* Houtt.
247		*Rumex microcarpus* Campd.
248		*Rumex obtusifolius* L.
249	Pontederiaceae	*Pontederia vaginalis* Burm. f.
250		*Pontederia korsakowii* (Regel & Maack) M.Pell. & C.N.Horn
251	Portulacaceae	*Portulaca oleracea* L.
252	Potamogetonaceae	*Potamogeton distinctus* A. Benn.
253	Primulaceae	*Androsace umbellata* (Lour.) Merr.
254		*Lysimachia candida* Lindl.
255		*Lysimachia christinae* Hance
256		*Lysimachia grammica* Hance
257	Pteridaceae	*Pteris multifida* Poir.
258	Ranunculaceae	*Ranunculus chinensis* Bunge
259		*Ranunculus japonicus* Thunb.
260		*Ranunculus sceleratus* L.
261		*Ranunculus sieboldii* Miq.
262	Rosaceae	*Duchesnea indica* (Andrews) Focke
263		*Potentilla supina* L.
264		*Rosa multiflora* Thunb.
265		*Rubus coreanus* Miq.
266		*Rubus parvifolius* L.
267	Rubiaceae	*Galium spurium* L.
268		*Paederia foetida* L.
269		*Rubia cordifolia* L.
270		*Serissa japonica* (Thunb.) Thunb.
271	Salviniaceae	*Azolla pinnata* subsp. *asiatica* R. M. K. Saunders & K. Fowler
272		*Salvinia natans* (L.) All.
273	Saururaceae	*Houttuynia cordata* Thunb.
274	Solanaceae	*Solanum japonense* Nakai
275		*Solanum lyratum* Thunb.
276		*Solanum nigrum* L.
277		*Solanum villosum* Mill.
278		*Lycium chinense* Mill.
279	Typhaceae	*Typha orientalis* C. Presl

280	Urticaceae	*Boehmeria nivea* (L.) Gaudich.
281		*Pouzolzia zeylanica* (L.) Benn. & R. Br.
282	Verbenaceae	*Verbena officinalis* L.
283	Violaceae	*Viola philippica* Cav.
284	Vitaceae	*Ampelopsis glandulosa* (Wall.) Momiy.
285		*Causonis japonica* (Thunb.) Raf.
286		*Vitis bryoniifolia* Bunge
287	Zygnemataceae	*Spirogyra communis* (Hass.) Kutz.
